# Draft genome sequence of Moroccan camelpox vaccine strain

**DOI:** 10.1128/mra.00869-23

**Published:** 2024-05-10

**Authors:** Z. Bamouh, Z. Elkarhat, J. Hamdi, S. Fellahi, K. Omari Tadlaoui, O. Fassi-Fihri, M. Elharrak

**Affiliations:** 1 R&D, MCI Santé Animale, Mohammedia, Morocco; 2 Department of Microbiology, Immunology and Contagious Diseases, Institute of Agronomy and Veterinary Hassan II, Rabat, Morocco; Katholieke Universiteit Leuven, Leuven, Belgium

**Keywords:** complete genome sequencing, camelpox virus

## Abstract

Prevention and control of camelpox can be achieved by efficient vaccination. A limited number of homologous attenuated vaccines have been commercialized. In this study, we report the draft genome sequence of camelpox virus vaccine strain “CAMPOX vaccine” after 175 passages of attenuation in Vero cells.

## ANNOUNCEMENT

Camelpox is a viral contagious disease of camels causing huge economic loss to the camel industry (1). Camelpox virus (CMLV) is a double-stranded DNA virus belonging to *Orthopoxvirus* genus of the *Poxviridae* family, with large genomes, ranging in length from 135 to 350 kbp (2–5).

The CMLV strain “CPVM08” was isolated from dry crusts obtained from camels during a 1984 outbreak in southern Morocco (6). The CPVM08 was propagated in Vero cells using a multiplicity of infection of 0.01 and harvested after 2–3 days of incubation at 35°C (7). The resulting viral suspension was titrated and inoculated for the second time in Vero cells. In total, 175 cell passages in Vero cells were performed to attenuate the CPVM175 strain (7). The vaccine administered by subcutaneous route is safe and affords protection to camels against virulent CMLV (7).

Two hundred nanogram of genomic DNA was extracted from the attenuated vaccine strain using the Isolate II genomic DNA kit (Bioline, UK) according to the manufacturer’s instructions (8). Library preparation was carried out using the Nextera XT DNA Library Prep kit (Illumina Inc., San Diego, CA, USA) according to the manufacturer’s protocol. Genome sequencing of the attenuated strain was carried out by the Eurofins Genomics Company, using Genome Sequencer Illumina NovaSeq 6000 S1 PE150 XP platforms.

Trimmomatic was used for the read quality trimming. The raw reads were subjected to quality (quality score threshold Q20) and length (above 40 nucleotides) trimming steps, and then sequencing adapter sequences were removed. This produced 27,429,972 total reads, 438,387 (1.6%) low-quality reads, 350,103 (1.3%) single reads, and 26,641,482 (97.1%) high-quality reads. The trimmed reads were assembled using CLC genomics v12 with the default parameters (CLC parameters: automatic determination of the word and bubble sizes with no scaffolding) using 2 kbp as the minimum contig size.

The draft genome of CML MOR P175 contains one contig comprising 191,707 bp and a G+C content of 32.94%, with an average coverage of 400×.

The assembly was annotated using Prokka annotation tool with default parameters (9). One copy of the duplicated reads is kept for father analyses; the others are discarded. The base quality recalibration is done using GATK modules 3.7 (10, 11).

The genomic sequence of the CPV M175 strain was compared by BLASTN to other complete CMLV genomic sequences available in the National Center for Biotechnology Information (NCBI) database. The CML MOR P175 showed 99.92% identity on the nucleotide level with CMPV-CMS virus isolated in Iran (AY009089.1) and CMLV strain 0408151 v (KP768318.1), 99.85% identity with CMLV M-96 isolated in Kazakhstan (AF438165.1), 99.67% identity with Negev 2016 strain isolated in Israel (MK910851.1) and a strain isolated in the UAE (MZ300859.1) (12–14).

The CMLV sequence reported here is the first available full CMLV genome from Morocco/Africa and will enrich the existing data of CMLV sequences.

## Data Availability

The whole-genome sequence of the CPV M175 was submitted to NCBI GenBank and is available under accession number OQ849768. The Illumina reads are available in the SRA under the accession number PRJNA1001289.
